# The Effect of Conflicting Pressures on the Evolution of Division of Labor

**DOI:** 10.1371/journal.pone.0102713

**Published:** 2014-08-05

**Authors:** Heather J. Goldsby, David B. Knoester, Benjamin Kerr, Charles Ofria

**Affiliations:** 1 Department of Biology, University of Washington, Seattle, Washington, United States of America; 2 Department of Microbiology and Molecular Genetics, Michigan State University, East Lansing, Michigan, United States of America; 3 Department of Computer Science and Engineering, Michigan State University, East Lansing, Michigan, United States of America; 4 BEACON Center for Evolution in Action, Michigan State University, East Lansing, Michigan, United States of America; University of Exeter, United Kingdom

## Abstract

Within nature, many groups exhibit division of labor. Individuals in these groups are under seemingly antagonistic pressures to perform the task most directly beneficial to themselves and to potentially perform a less desirable task to ensure the success of the group. Performing experiments to study how these pressures interact in an evolutionary context is challenging with organic systems because of long generation times and difficulties related to group propagation and fine-grained control of within-group and between-group pressures. Here, we use groups of digital organisms (i.e., self-replicating computer programs) to explore how populations respond to antagonistic multilevel selection pressures. Specifically, we impose a within-group pressure to perform a highly-rewarded role and a between-group pressure to perform a diverse suite of roles. Thus, individuals specializing on highly-rewarded roles will have a within-group advantage, but groups of such specialists have a between-group disadvantage. We find that digital groups could evolve to be either single-lineage or multi-lineage, depending on experimental parameters. These group compositions are reminiscent of different kinds of major evolutionary transitions that occur within nature, where either relatives divide labor (fraternal transitions) or multiple different organisms coordinate activities to form a higher-level individual (egalitarian transitions). Regardless of group composition, organisms embraced phenotypic plasticity as a means for genetically similar individuals to perform different roles. Additionally, in multi-lineage groups, organisms from lineages performing highly-rewarded roles also employed reproductive restraint to ensure successful coexistence with organisms from other lineages.

## Introduction

In *The Origin of Species* [1], Darwin used examples of artificial selection to lay the groundwork for his natural selection analog. More recently, experiments using artificial selection have greatly increased our understanding of both short- and long-term evolutionary processes [2]. Indeed, while the role of group selection in natural settings is a controversial topic [3], [ 4], artificial group selection experiments have demonstrated that selection at the level of the group can produce targeted evolutionary responses [2], [ 5]–[8]. For example, in a classic group selection experiment, Wade preferentially selected groups of flour beetles (*Tribolium castancum*) for both large and small group sizes [7]. After nine group-selection events, the beetles selected for large group size averaged 178 individuals, while the beetles selected for small group size averaged only 20 individuals. More recently, Swenson *et al.* have shown that artificial group selection can produce soil systems supporting an increased plant biomass [5], and aquatic ecosystems with a specific pH [6].

Of particular interest are instances of *antagonistic multilevel selection*, where the between-group pressure (i.e., whether groups thrive) conflicts with the within-group pressure (i.e., whether individuals within the group thrive). For example, within Wade's study, there are indications that selecting groups for larger population sizes involved antagonistic multilevel selection pressures. Cannibalism would provide an individual with a within-group advantage, but groups of cannibals would likely have a smaller population size and thus a between-group disadvantage. Specifically, when groups with larger population sizes were preferentially propagated, cannibalism was a group-level liability. Indeed, individuals exhibited lower rates of cannibalism when large groups were selected for [8]. Conversely, cannibalism rates were higher when groups with smaller populations were preferentially propagated.

Antagonistic multilevel pressures are frequently observed in groups that exhibit division of labor within nature, where there is a within-group pressure to specialize on the role with the highest reward and a between-group pressure to perform a diverse suite of tasks. These groups, which vary in scale and complexity, include a host cell and its mitochondria [9], the cells of a multicellular organism [9], [ 10], insects within colonies of eusocial arthropods [11]–[15], and even humans within societies [16], [ 17]. Even though these groups are the epitome of high-level functionality, when the within-group pressure asserts itself, it can produce ills such as mitochondrial and nuclear genome interactions that result in plants that do not produce viable pollen [18], cancer within multicellular organisms [19], defector insects within eusocial colonies [20], and criminals within human societies [21]. While we present our work in terms of multilevel selection [22], [ 23], such evolution can also be viewed from an inclusive fitness perspective [24]–[26] (where a focal individual's fitness can be decomposed into the effects of its phenotype on individuals within its group, including itself, weighted by its relatedness to these individuals). Ideally, a complete analysis of antagonistic multilevel selection pressures would include open-ended evolutionary dynamics in a system that has rapid generation times and is tractable enough to facilitate an exploration of mechanisms.

For this study, we use the Avida digital evolution platform [27]. Digital evolution is a form of experimental evolution, where organisms are self-replicating computer programs that evolve in a user-defined computational environment and are subject to mutations and natural selection. These *digital organisms* execute instructions in their genome to metabolize resources in the environment, interact with neighboring organisms, and self-replicate. Digital evolution has previously been used to study topics in evolutionary biology ranging from the origin of complex features [28], modularity [29], leader election [30], [ 31], altruism [32]–[35], and division of labor [36]–[38]. Digital evolution fills a unique niche in the study of evolutionary phenomena. In contrast to analytical models and simulations, the digital approach is an open-ended instance of evolution. However, in contrast to experimental evolution with organic systems, digital evolution enables us to study evolution over many more generations, and with unparalleled experimental control and automated data collection, which facilitates the exploration of mechanisms employed by digital organisms. For this study, digital evolution enables us to manipulate factors that may affect the course of evolution within a group-structured system. Specifically, we vary the degree of antagonism among group and individual selection pressures, the methods by which groups are formed, and how group members interact.

We first examine the evolutionary trajectories of groups that exhibit division of labor when individual roles have different fitness benefits. We begin these experiments with several isolated groups of genetically-identical ancestor organisms, and allow these organisms to differentiate via mutation. In this paper, we address the question: Given both a between-group pressure to perform a variety of tasks, and a within-group pressure to perform the task with the highest associated fitness benefit, under which conditions will groups of organisms evolve to perform a diverse suite of tasks? Second, when division of labor evolves, we explore how individuals fill different roles, especially roles that have a within -group disadvantage. Within nature, we observe two common strategies: (1) Organisms may form single-lineage groups (i.e., groups of closely-related individuals), where some members perform less rewarded roles via phenotypic plasticity (e.g., workers in eusocial colonies and somatic cells within multicellular organisms); or (2), organisms may coexist within genetically heterogeneous groups (e.g., a host cell and its mitochondria), where different genetic lineages occupy distinct niches and are co-transmitted to the next generation. Digital evolution enables us to examine the conditions under which single-lineage or multi-lineage groups of organisms are favored by selection. We then explore the mechanisms by which individuals within single-lineage and multi-lineage groups coordinate to perform different roles.

## Avida Digital Evolution System

Within Avida, digital organisms compete for space in their environment. Each digital organism is a fully functional computer program, arranged as a circular list of instructions, and a virtual CPU that executes the instructions. The instructions in an organism's genome determine the organism's behavior, including its ability to sense and change properties of its environment. Because organisms are *self-replicating*, the genome itself must contain the sequence of instructions needed to create an offspring. When an organism replicates, a neighboring location is selected from the environment, and any previous inhabitant of the target location is replaced (killed and overwritten) by the offspring. Genomes are subject to random mutations (substitutions, insertions, and deletions) during the replication process, leading to offspring that are genetically distinct from their parents.

The genomes of digital organisms can include a variety of different instructions drawn from the Avida instruction set. These instructions include those for basic computational tasks (e.g., addition, subtraction, and bit-shifts), controlling execution flow, communication, environmental interaction, and self-replication (these instructions are described in detail in [27]). The standard Avida instruction set is designed so that any combination of instructions is a syntactically correct program, albeit one that may not perform any meaningful computation [39]. In this study, the instruction set also included several instructions that were developed to facilitate distributed problem solving [40]; these instructions enable organisms to send messages to one neighbor (an adjacent organism on a toroidal grid), to all of their neighbors, to retrieve a message, and to access their spatial position (i.e., x-y coordinates) within the group. Additionally, organisms were able to transmit epigenetic information to their offspring. Specifically, numerical values stored in one particular register were not erased during replication and could be used to facilitate coordination.

The *metabolic rate* of a digital organism determines the relative rate at which an organism's virtual CPU executes the instructions in its genome. For example, an organism with a metabolic rate of 10 will, on average, execute instructions twice as quickly as one with a metabolic rate of 5. Because organisms self-replicate and compete for space, an organism with a higher metabolic rate will generally grow to dominate the world, all else being equal. Within Avida, an organism can perform a *task* (or exhibit a specific phenotype) in order to consume resources that will increase its metabolic rate. For most of the experiments described in this study, we use five mutually-exclusive logic operation tasks. Specifically, to receive a reward, an organism must perform a bitwise Boolean logic operation on 32-bit integers. The tasks are configured such that the organisms that perform them receive unequal rewards. Specifically, if an organism performs task NOT or NAND (the simplest tasks), then its metabolic rate is doubled. If an organism performs task AND or ORNOT, its metabolic rate is tripled. If an organism performs task OR (the most complex task in this environment), its metabolic rate is quadrupled. An organism can only receive a reward for performing one task. Thus, an organism that performed task NAND could not subsequently receive a reward for performing task ORNOT.

**Figure 1:**
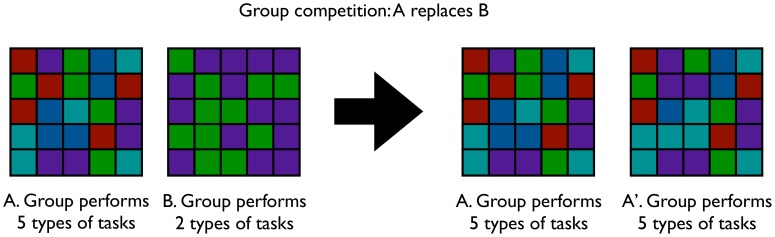
Depiction of the group replication process. Group A performs a more diverse suite of tasks than group B. Thus, when the groups compete in the tournament, group A is preferentially replicated and a mutated copy of group A replaces group B. Colors indicate different phenotypes.

To study multilevel selection in Avida, we divided the digital organisms into distinct *groups* that compete. An Avida world consists of 400 groups. In this study, groups compete and are replicated via tournament selection. For *tournament selection*, every 100 updates, groups that exhibit division of labor have a fecundity advantage (Figure 1). (An *update* is the unit of experimental time in Avida corresponding to an average of 30 virtual CPU instructions per organism.) We refer to the time period between tournament events in which we compete the groups as the *inter-tournament period length*. Each round of between-group selection consists of 400 tournaments, where the groups in each tournament are selected at random with replacement. Within each tournament, the group that performs the greatest variety of types of tasks is replicated to the next group-generation (ties are broken randomly). The strength of tournament selection varies with the size of each tournament. For example, a tournament size of five groups results in a stronger between-group pressure than a tournament of two groups. We performed most of the experiments described in this paper with tournaments of size 5. However, to more fully understand how tournament size affects division of labor, we also included additional treatments in which groups compete in tournaments that vary in size from 2 to 20. (Text S1, Figure S1.)

Within each group, organisms are still able to self-replicate and experience mutations. Organisms are allocated CPU cycles on the basis of their metabolic rate compared to the population. However, organisms directly compete with their group members for space. Specifically, when an organism replicates, it replaces a member of its group. As a result, it is possible to establish either single-lineage or multi-lineage groups. Since all constituent organisms are copied when we replicate a group, an individual's long-term survival is dependent not only on its ability to out-compete its neighbors for the limited space available in its group, but also on the collective ability of the group to out-compete other groups.

## Results

First, we examine how various combinations of multilevel selection pressures affect the diversity of the suite of tasks performed by groups of organisms. Next, we vary key parameters and observe their effect on the evolution of division of labor. Finally, we explore the mechanisms used by individuals within successful groups to coordinate their roles.

### How do groups of organisms evolve to respond to multilevel selection pressures?

In our central experiment, we investigate whether division of labor evolves under treatments that vary the within-group and between-group pressures. Specifically, we define four treatments: Within includes within-group pressures only; Between includes between-group pressures only; Both includes both within- and between-group pressures; and None includes neither within-group nor between-group pressures (a control). (Refer to Materials and Methods for details.) We predict that if between-group selection is necessary for evolving division of labor, then treatments Between and Both should evolve to exhibit a wider range of tasks than treatments Within and None. Moreover, if treatment Both performs a wider range of tasks, then we are also able to provide evidence that the between-group selection pressure for division of labor is sufficient to counteract the within-group selection pressure to perform the most highly-rewarded role.

**Figure 2:**
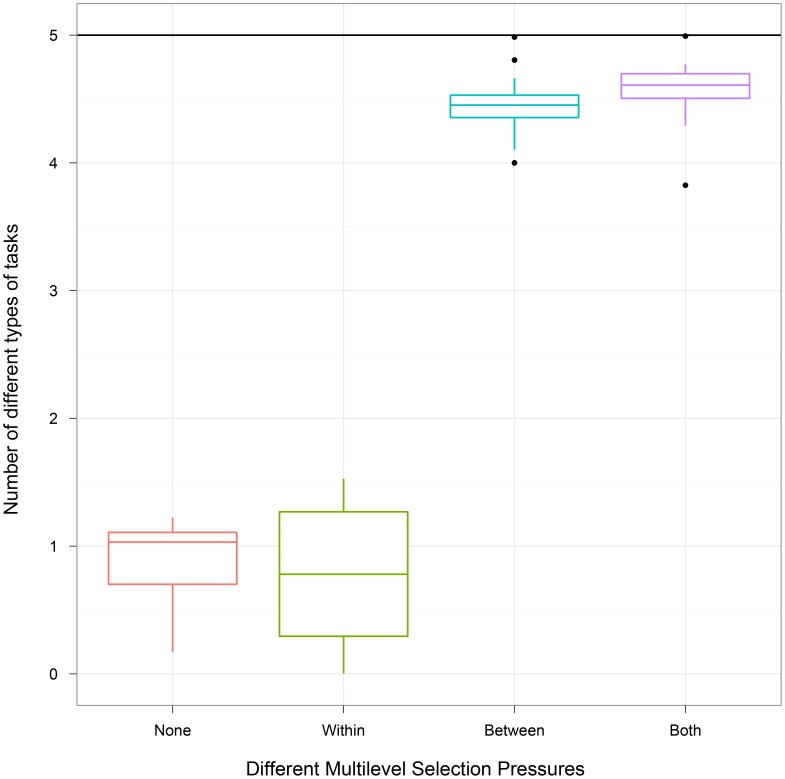
Varying within-group and between-group pressures. We report the mean number of different types of tasks performed by groups of organisms in each treatment. Values are taken over 30 replicates in an environment where each organism can perform one of five different logic tasks. Thus, the maximum number of different types of tasks that can be performed by a group (indicated by a black horizontal line) is 5. The treatments evolved with between-group selection pressures outperform the treatments evolved without between-group selection pressures. These results are evidence that, in this environment, the between-group component of the selection pressures is necessary to produce groups of organisms that succeed at performing a wide range of tasks.

Figure 2 depicts the number of unique types of tasks performed by groups of organisms for these four treatments. Treatments that include the between-group pressure (Both and Between) evolved to perform a wider range of tasks than those without the between-group pressure (Kruskal-Wallis multiple comparison, 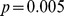). Summary statistics for this and all future treatments can be found in Table 1. The mean of the None treatment is close to one. This result occurs because the None treatment removes differential task pressures by rewarding all tasks evenly. However, an organism that performs any task outcompetes all organisms that perform no tasks. Counterintuitively, the mean of the Both treatment exceeds that of the Between treatment (though this is a non-significant difference). Multilevel selection pressures exist in a continuum of conflict ranging from complete alignment to complete opposition of within-group and between-group pressures. The Both treatment explores one form of antagonistic pressures present within division of labor systems, where antagonism results from a within-group pressure to perform the most highly-rewarded tasks and a between-group pressure to perform all tasks. However, within the Both treatment, the multilevel selection pressures are not completely in conflict – An individual always receives a reward for performing a task that is beneficial for the group. We performed an additional treatment, where the pressures were more antagonistic (i.e., all tasks are individually detrimental to varying degrees). For this treatment, we rewarded the tasks as follows: NOT (0.25), NAND (0.25), AND (0.50), ORN (0.50), and OR (0.75). In this case of extreme antagonism, the mean number of unique tasks performed was 3.66
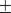
0.15 (standard error) in the Both treatment, whereas the mean number of unique tasks performed in the Between treatment is 4.46
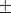
0.04 (Wilcox Multiple Comparison, 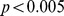). We more fully explore the effects of modifying the within-group pressure in Text S2 and Figures S2 and S3.

**Table 1:** Summary statistics for experimental treatments.

Experiment	Treatment	Performance	Levenschtein Distance
		Mean 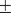 Standard Error	Mean 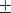 Standard Error
Multilevel Selection Pressures			
	Within	0.77 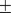 0.09	-
	Between	4.46 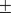 0.04	45.67 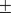 0.53
	Both	4.51 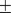 0.06	27.98 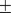 13.11
	None	0.89 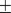 0.06	-
Inter-Tournament Period Length			
	50-update	4.71 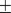 0.02	41.46 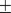 7.90
	100-update*	4.51 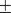 0.06	27.98 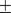 13.11
	250-update	4.18 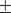 0.06	8.03 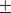 3.10
	500-update	3.85 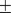 0.06	-
Migration Rate			
	0%*	4.51 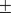 0.06	27.98 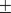 13.11
	5%	2.99 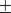 0.06	-
	10%	2.46 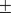 0.05	-
	20%	2.05 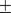 0.04	-
Propagule Size			
	25*	4.51 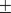 0.06	27.98 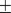 13.11
	1-S	3.02 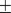 0.13	-
	2-S	3.65 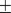 0.11	-
	3-S	4.24 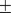 0.04	0.04 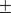 0.12
	5-S	4.43 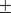 0.04	0.30 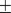 0.09
	15-S	4.57 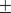 0.07	9.61 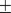 5.81
	25-S	4.51 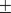 0.05	15.30 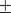 4.11

Mean and standard error are computed over 30 replicates at the final time point. An asterisk (*) denotes that the data from the Both treatment from our original experiment was used for the particular treatment.

### How is division of labor affected by inter-tournament period length, migration rate, and propagule size?

Three factors that have the potential to disrupt the evolution of division of labor are: (1) the frequency at which groups compete compared to the life-span of individual organisms, (2) the degree to which established groups are isolated from others, and (3) the method by which new groups are formed. Here we explore the effect of these factors on division of labor. For all of these experiments, we maintain the same multilevel selection pressures used for the Both treatment.

#### 1. Inter-tournament period length

The inter-tournament period length alters the relative strength of between-group selection as compared to within-group selection. Specifically, as the duration of this period increases, the force of between-group selection is weakened; conversely, as the duration of this period shrinks, the force of between-group selection is strengthened. Given the effect of the between-group pressure, the amount of division of labor present within the groups should decline as the duration of the period between tournaments increases. To test this prediction, we performed an experiment where treatments had inter-tournament periods of 50, 100, 250, and 500 updates. Within our central experiment, the Both treatment used an inter-tournament period of 100 updates.

**Figure 3:**
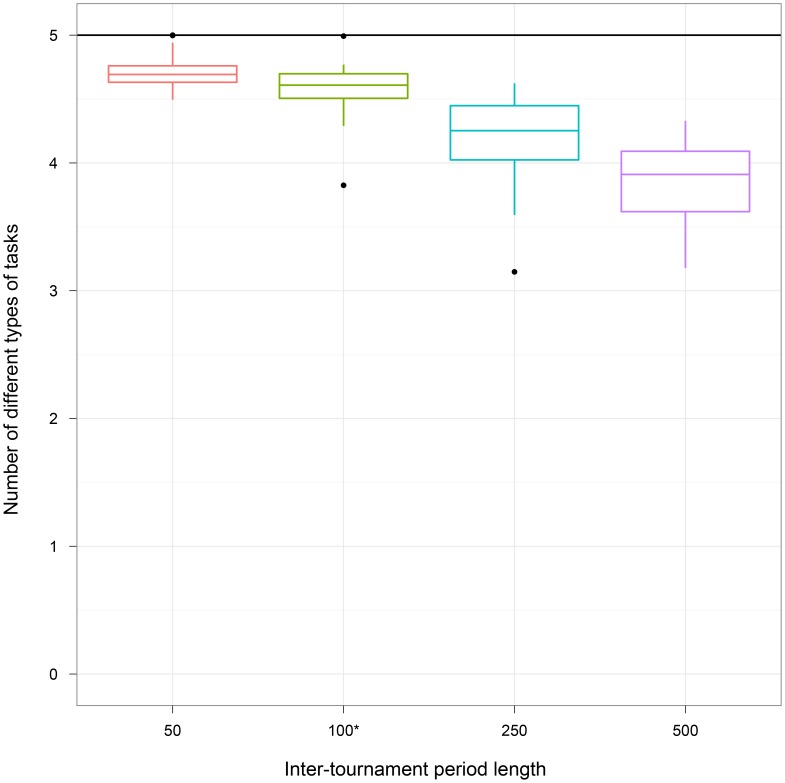
Varying the inter-tournament period length. We report the number of different types of tasks performed by groups of organisms in each treatment, where treatments had different between-group selection intervals. The maximum number of different types of tasks that can be performed by a group (indicated by a black horizontal line) is 5. The asterisk (*) indicates the Both treatment. As the inter-tournament period length increased, the amount of division of labor exhibited by the groups decreased. However, inter-tournament period length does not have a qualitatively large effect.

Figure 3 depicts the results of this experiment (see Table 1 for summary statistics). In general, as the inter-tournament period increases, the mean number of unique types of tasks performed by group members decreases. The 50-update and 100-update treatments are both significantly different than the 250-update and 500-update treatments, but not from each other (Kruskal-Wallis multiple comparison, 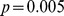). While increasing the inter-tournament period length decreases the amount of division of labor evolved, it does not have a large effect. Specifically, we observe that changing from a competition period of 50 updates to one ten times longer (500 updates) results in the loss of less than one task on average (4.71
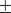
0.02 for the 50-update treatment and 3.85
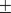
0.06 for 500-update treatment). In further analyses, we also determined that the organisms evolved to time their task performance to coincide with the inter-tournament period length and performance decreased when we transplanted organisms evolved with a short period into a long period and vice versa (Table S1).

#### 2. Migration

A second factor that has the potential to disrupt the ability of groups of organisms to evolve to perform a wide range of tasks is migration. Within Avida, migration occurs as part of the individual replication process. Specifically, a configurable migration rate determines the probability that an offspring organism will be placed into a group different than that of its parent. For our central experiment, the migration rate was set to 0% (no migration), ensuring that all offspring were born into the same group as their parent. As the migration rate increases, the cohesiveness of the group declines, since organisms may not be placed in groups with kin or other mutualistic clades that their ancestors evolved with. Thus, our prediction is that higher migration rates will result in a decline in the amount of division of labor exhibited by evolved groups. To study the effects of migration, we performed an experiment in which treatments had migration rates of 0%, 5%, 10%, and 20%.

**Figure 4:**
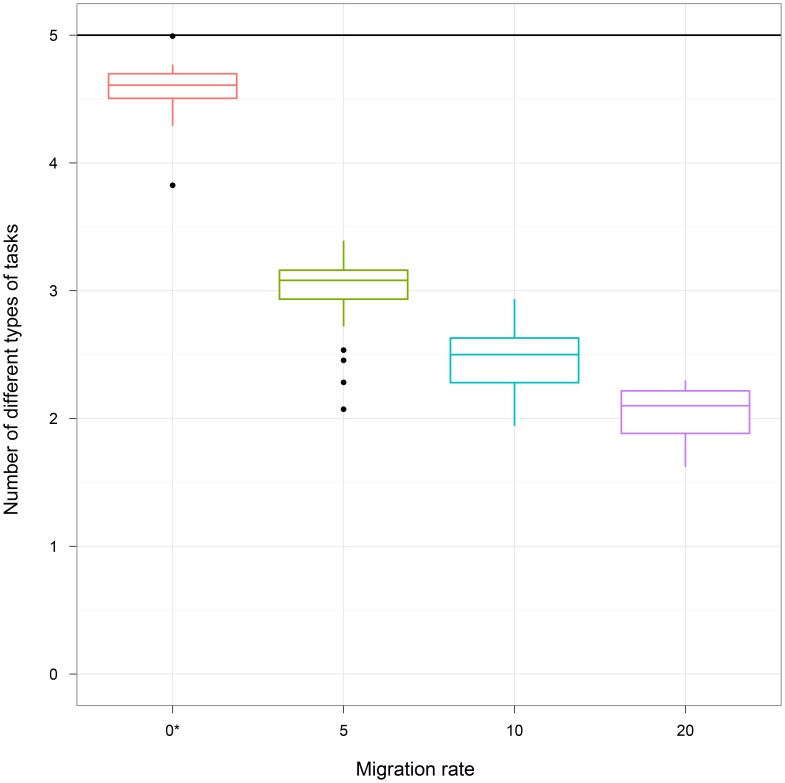
Varying migration rates. We report the number of different types of tasks performed by groups of organisms in each treatment, where treatments had different migration rates. The maximum number of different types of tasks that can be performed by a group (indicated by a black horizontal line) is 5. The asterisk (*) indicates the Both treatment. As migration rate increased, the number of types of tasks performed by the groups substantially decreased.

Figure 4 depicts the results of our migration experiment (see Table 1 for summary statistics). 0% migration is significantly different than all other treatments and 5% migration is significantly different than 20% (Kruskal-Wallis multiple comparison, 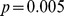). Even a small probability of migration has a large effect on the ability of the group members to perform a wide range of tasks. Specifically, changing from a migration rate of 0% to a migration rate of 5% causes a loss of 1.523 tasks on average, and the number of tasks continues to drop as migration rate is increased further.

#### 3. Propagule Size

There are several different methods by which we can generate an offspring group from a parent. These methods include creating a copy of the parent group, mixing individuals from two or more groups, and sampling individuals from a successful group to form the offspring group [6], [41]–[43]. In our central experiment, to create a new group, we copied all the digital organisms from a single successful group. However, it is possible that varying how a new group is created may affect the ability of its constituent organisms to coordinate roles and thus to perform division of labor. To ascertain how other approaches to group creation affect division of labor, we performed an additional experiment in which we created new groups by sampling organisms from the original group with replacement. Additionally, we varied the *propagule size*, which is the number of organisms used to seed the new group, from 1 to 25. Groups created with a propagule of size 25 differ from the Both treatment in our central experiment in that, as a result of sampling, they may not include all participants from the source group.

**Figure 5:**
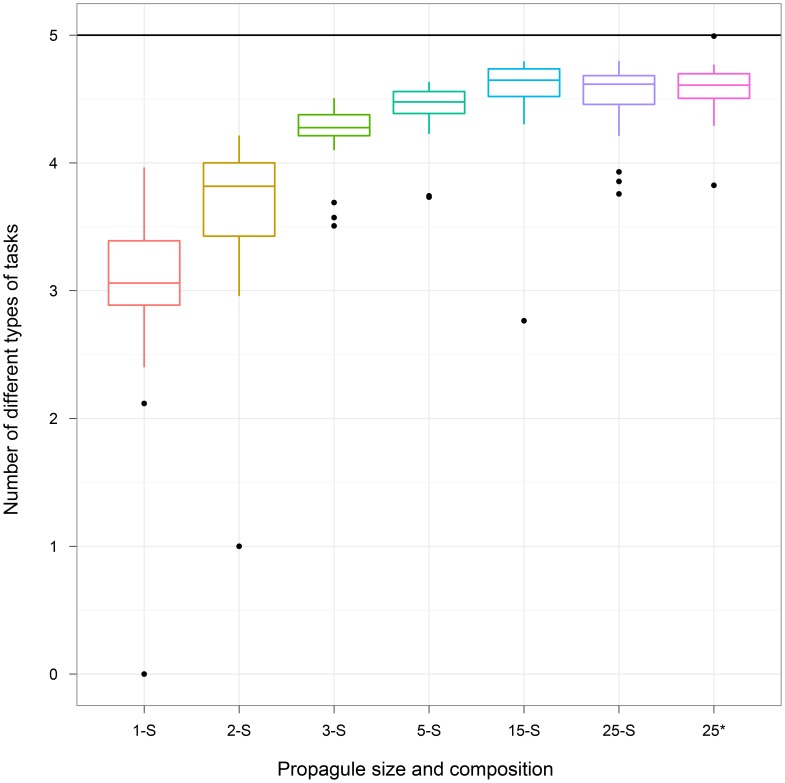
Varying propagule size. We report the number of different types of tasks performed by groups of organisms in each treatment, where treatments had different propagule sizes. The maximum number of different types of tasks that can be performed by a group (indicated by a black horizontal line) is 5. The asterisk (*) indicates the Both treatment. Small propagule sizes (1–3) correspond to a lower diversity of tasks within the group. However, the performance of propagule sizes of 5 or more are not significantly different from each other. Here “S” indicates that propagules were created using sampling with replacement.

Figure 5 depicts the results of our propagule experiment (see Table 1 for summary statistics). At small propagule sample sizes (1–3), group members perform a less diverse suite of tasks (Kruskal-Wallis multiple comparison, 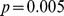). One explanation for this phenomenon is that sampling may select inferior group members for propagation. Once the propagule size increases to 5, however, the number of unique types of tasks evolved is indistinguishable from larger propagule sizes.

Overall, these experiments demonstrate that groups of organisms that perform division of labor are able to evolve under a variety of conditions. Such groups of organisms evolve when placed under multilevel selection pressures that range from antagonistic to aligned (see Text S2 for further details). Additionally, groups of organisms that exhibit division of labor are favored over a broad range of evolutionary parameters, including different inter-tournament period lengths and propagule sizes three or above. However, the amount of division of labor within evolved groups of organisms is substantially less when members migrate between groups.

### What strategies do organisms use to successfully address the antagonistic multilevel selection pressures?

While these results demonstrate that division of labor evolves under a variety of multilevel selection regimes, they tell us little about *how* the organisms acquire their roles given the disparate fitness benefits. Within our experiments, groups initially consisted of genetically-identical organisms, but over time mutations accrued as part of the individual replication process could produce either single or multi-lineage groups. For this portion of the study, we focus on treatments where the mean number of unique tasks exceeds four. First, to determine whether the groups were single-lineage or multi-lineage, we measured genotypic variation within the groups. To do so, we calculated the mean *Levenshtein distance* (i.e., the minimum number of insertions, deletions, and substitutions necessary to convert one genome into another [44]) of random pairs of organisms within each group. A potentially confounding factor for this analysis was genetic diversity introduced as the result of deleterious mutations (e.g., a mutation that caused task loss) introduced into the genome of offspring organisms. Such genetic diversity is likely not adaptive. To eliminate this effect, prior to calculating the Levenshtein distance, we subjected all of the organisms to a 1,000 update *ecological period* during which no mutations occurred. The ecological period purged the groups of genotypes with lower fitness. During this period, we continued to compete the groups every 100 updates for a total of 10 tournaments.

Table 1 provides the mean Levenshtein distance and standard error within groups for the various treatments whose performance exceeded four tasks. Based on these data, we make several observations. First, these treatments have produced both single-lineage and multi-lineage groups. The Levenshtein distances vary from a mean of 0.04 (treatment 3-S), which indicates a single-lineage group, to 45.67 (treatment Between), which indicates a multi-lineage group.

Second, genetic variation is inversely proportional to the inter-tournament period length. Replicates with a tournament length of 50 updates had significantly more genetic variation than replicates with tournament lengths of 250 (Kruskal-Wallis multiple comparison, 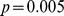). These data suggest that decreasing the between-group selective pressure by extending the duration of the tournament not only reduces the number of distinct types of tasks performed by group members, but also reduces the amount of genetic variation present.

Third, larger propagule sizes increase the amount of genetic diversity present within groups without decreasing overall number of types of tasks performed by members of the groups. Groups of organisms evolved with small propagule sizes adopted single-lineage strategies, whereas groups of organisms evolved with larger propagule sizes exhibited strategies that relied on genetic diversity (e.g., the Levenshtein distance for a sampled propagule of size 3 is 0.04
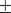
0.12 compared to 15.30
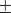
4.11 for a propagule of size 25). We further explored these results by analyzing the number of types of tasks performed and genetic diversity of individual replicates within the treatments. We consider a replicate to be genetically heterogeneous if it had a Levenschtein distance greater than 25. (The ancestor organisms were 100 instructions long, thus a Levenschtein distance of 25 means that on average approximately 25% of the genomes differ.) For these treatments, only 15-S, 25-S, and 25 contained replicates that were genetically heterogeneous. This result strongly supports the observation that only replicates with larger propagule sizes exhibited strategies that relied upon genetic diversity. Intuitively, this is to be expected because organisms within a group with a small propagule size could not rely on representatives of each lineage to be copied during a group replication event. Within the three treatments that had replicates that exhibited both single lineage and multi-lineage strategies, the amount of division of labor exhibited by the replicates that used single-lineage strategies exceeded that of the replicates that used multi-lineage strategies. The mean amount of division of labor (number of different types of tasks performed) for the multi-lineage replicates were: 4.22
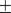
0.16 for 15-S, 4.30
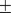
0.02 for 25-S, and 4.33
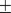
0.16 for 25. In contrast, for the single-lineage replicates, they were: 4.56
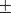
0.08 for 15-S, 4.61
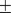
0.05 for 25-S, and 4.60
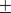
0.04 for 25.

A second effect captured within these results is how sampling affects genetic diversity. In the original Both treatment, all group members were copied. This treatment (25*) has the highest amount of genetic diversity, which surpasses that of a propagule size of 25 created by sampling from the original group (25-S). This result indicates that, similar to smaller propagule sizes, sampling reduces reliance on multi-lineage strategies, since it decreases the probability that a member from each lineage will be selected as part of the propagule.

Finally, upon closer examination, we noted that the Levenshtein distance of the replicates for the Both treatment indicated a bifurcated strategy: The genetic variation of some replicates collapsed to negligible amounts, whereas the genetic variation of other replicates remained quite high. A 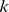-means clustering of Levenshtein distances revealed that 18 replicates were clustered into a group with a mean distance of 4.214 instructions, 11 replicates were clustered into a group with a mean of 33.060 instructions, and 1 replicate, an outlier, had a distance of 399.937 instructions. Some replicates exhibited a wide-range of tasks within the context of single-lineage groups, whereas other replicates maintained multi-lineage groups.

These results demonstrate the ability of Avida to evolve strategies that reflect those commonly observed in nature. Moreover, they highlight the benefit of the open-ended approach to studying this question. The experimental design is such that, depending on the balance of pressures, either a single-lineage or a multi-lineage strategy may emerge.

### What mechanisms are used by organisms to preserve diversity within groups?

In this study, groups maintained two forms of diversity. First, both single and multi-lineage groups maintained phenotypic diversity, in that the organisms performed a diverse suite of tasks. Second, multi-lineage groups also maintained genotypic diversity. Here we investigate the strategies used by groups to maintain both forms of diversity.

#### Phenotypic diversity

For both single-lineage and multi-lineage strategies, organisms evolved to coordinate roles and maintain phenotypic diversity. Within single-lineage groups, such mechanisms were critical for organisms to differentiate roles and thus achieve division of labor. Evolved mechanisms could rely on either stochasticity or phenotypic plasticity. Previous studies using Avida have demonstrated the ability of organisms to evolve phenotypic plasticity [33] using either environmental inputs or execution flow to differentiate, so the underlying mechanism can clearly be evolved by the organisms.

Organisms were provided with several coordination instructions that, in principle, could be used to differentiate roles. To understand how the emergent behavior of a group of organisms is influenced by the instructions within its constituent genomes, we conducted *knockout analyses*, where we replaced one or more instructions in all of the genomes in a group with a neutral instruction. We then subjected the organisms to an ecological period and monitored their behavior. If the group members performed fewer types of tasks when a coordination instruction was removed, then we can conclude that the knocked-out instruction contributed to the division of labor exhibited by the group.

We performed three different sets of knockout analyses: (1) Knockouts of location-sensing capabilities (i.e., the get-cell-xy instruction); (2) Knockouts of messaging capabilities (i.e., the retrieve-msg instruction); and (3) Knockouts of the epigenetic information capability (i.e., the get-epigenetic instruction). For the control treatments, which underwent an ecological period, but did not experience any knockouts, the median number of types of tasks performed was 5 (the maximum) and the mean was 4.500. When we knocked out the ability to communicate using messaging, the median number of types of tasks performed remained at 5 and the mean dropped only slightly to 4.467, indicating that most of the organisms were not using messaging. When we knocked out location-sensing information, the median number of types of tasks performed dropped to 4 and the mean was 4.003, indicating that the organisms were making limited use of location information to coordinate roles. However, when we knocked out epigenetic information, the median number of types of tasks performed dropped to 3 with a mean of 3.2, thus indicating the organisms were making use of epigenetic information to coordinate roles.

In further analyses of the strategies employed by members of multi-lineage groups (Text S3 and Figures S4 and S5) and single-lineage groups (Text S4), we confirmed that organisms used epigenetic information to differentiate roles by passing values from parent to offspring that were used in subsequent computations. This behavior is similar to cellular differentiation in which parent cells pass state information to offspring cells, which they use to become increasingly specialized over time [45]. Within a multi-lineage group, each lineage may perform multiple tasks using epigenetic information. However, in general, it is the case that different lineages perform different subsets of tasks. Thus, within these groups, all lineages must be present to perform the full suite of tasks. One question that arises is how do lineages that perform more highly-rewarded tasks avoid replicating over other lineages? In other words, how is genetic diversity sustained within multi-lineage groups?

#### Genetic diversity

To understand how genetic diversity is maintained, we examine the behavior of groups of organisms evolved as part of the Both treatment. To maintain genetic variation within a group, organisms with different lineages must balance their rate of replication. If the rates of replication were greatly disparate, organisms within the most successful lineage would fix within the group, thus decreasing genetic diversity and the amount of division of labor exhibited by the group.

In Avida, an organism's rate of replication, 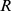, is defined as: 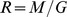, where 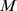 is metabolic rate and 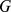 is gestation investment. The metabolic rate of a digital organism defines the number of virtual CPU cycles it is allocated per unit time, and is modified by the rewards of any tasks that the organism performs. An organism's *gestation investment* is the number of virtual CPU cycles that are expended to produce an offspring. The primary factor that affect an organism's gestation investment is the efficiency of replication (i.e., how many cycles it takes to produce an offspring). A genotype's rate of replication is considered to be the mean rate of replication of all the organisms that share that genotype.

As a first step, we verified that *genotypes* present within the same group had similar rates of replication. To perform this measurement, we selected the group from each replicate that exhibited the greatest amount of division of labor after being subjected to an ecological period of 1,000 updates. The variance in the replication rate of genotypes within groups is significantly smaller than the variance in the replication rate of genotypes among groups (permutation test using Anova F-statistic, 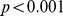). This result is consistent with the idea that the organisms are indeed evolving to equalize rates of replication among genotypes present within each group.

Thus far, we have confirmed that genotypes present within the same group share similar rates of replication. Additionally, we know that organisms within the group have different metabolic rates because they are performing different tasks. If organisms within a group are in fact balancing their respective replication rates, then we would expect to see a positive relationship between gestational investment and metabolic rate for *organisms* in the same group. To address this prediction, we recorded the gestational investment and metabolic rate of every organism in each sampled group over a period of 100 updates. We find that the slope of the least squares line for the (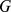, 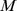) data of organisms within each group is always positive. One concern is that there may be a system-level constraint within Avida that forces all (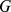, 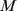) pairs to have the same relationship. To test whether the slope of this relationship varied among groups, we permuted the group membership of the organisms and recorded the sum of squares for the least squares linear fit for each group and summed this metric across groups. For all 1000 permutations, the total sum of squares was higher than that of the original data, suggesting that the null hypothesis that all groups have the same relationship between gestational investment and metabolic rate can be rejected. Consistent with the previous permutation test, this one also suggests that the balancing of replication rates is occurring within each group independently and does not reflect an inherent constraint of Avida.

To further establish that the relationship between gestational investment and merit did not result from an artifact of Avida, we also examined how the relationship degraded when the between-group pressure was removed. Specifically, for each selected group, we filled a population with copies of the same group. We ran this population for an additional 10,000 update period without the between-group pressure, but with mutations. After that, for a 100 update period, we recorded the gestational investment and metabolic rate of every organism. We compared the metabolic rate to gestational investment ratio of the organisms within the evolved population to those within the original groups. Over 85% of the organisms evolved to exhibit a higher ratio indicating that they were abandoning their balance of metabolic rate and gestational investment to become more fit. These data are evidence that the multilevel selection pressures, rather than an artifact of Avida, produce the positive covariance between gestational investment and metabolic rate. By balancing these factors, different genotypes within the same group maintain similar replication rates and thus avoid eliminating one another.

## Discussion

We have explored the evolution of organisms that experience multilevel selection pressures. We investigated a continuum of within-group and between-group pressures and demonstrated that it is possible to evolve groups of organisms that exhibit division of labor even when the within-group pressure to perform a highly-rewarded task counters the between-group pressure to perform a diversity of tasks. Additionally, we have explored how the performance of these groups of organisms is affected by the duration between group competitions (inter-tournament period length), migration rate, and method by which the groups are formed. From the multilevel perspective, these factors affect group homogeneity, thus affecting the balance of power between the within-group pressure and between-group pressure. From the inclusive fitness perspective, these factors affect the coefficient of relatedness among individuals within a group. We found that the amount of division of labor exhibited by groups of organisms was robust to various inter-tournament period lengths and propagule sizes over 3. However, even a small amount of migration substantially decreases the amount of division of labor exhibited by the groups.

Next we examined the mechanisms by which tasks were allocated among organisms within successful groups, in particular, how some members came to perform less rewarded roles. The groups of organisms exhibited both single-lineage and multi-lineage strategies. Organisms within both single-lineage and multi-lineage groups used phenotypic plasticity to differentiate roles. Additionally, within multi-lineage groups, mutualistic lineages balanced their fecundity to avoid replicating over one another.

Major transitions in evolution occur when formerly distinct individuals form a higher-level unit that functions as a single reproductive entity [46]. These transitions can be *fraternal*, where genetically similar individuals (i.e., close kin) differentiate to perform various tasks, or *egalitarian* in which formerly distinct organisms create a super-organism that replicates all of its genetic material [9], [47]. For example, fraternal transitions include single cells transitioning into multicellular organisms and solitary insects transitioning into eusocial colonies; egalitarian transitions include the “eukaryotic alliance between a host cell and its mitochondria” [9]. These transitions raise evolutionary questions regarding why formerly distinct individuals would cooperate with others and, once they did, how this arrangement persisted. Groups formed via fraternal and egalitarian transitions exhibit division of labor, where individuals within the groups perform different associated roles. Within our current experiments, we have observed groups of organisms evolving strategies that parallel the end results of the two types of transitions. Specifically, some groups of organisms evolved single lineage strategies in which genetic diversity was low and others evolved multi-lineage strategies with greater degrees of genetic diversity.

Fraternal and egalitarian major transitions must both address two central challenges: (1) the origination of differentiated roles, and (2) the persistence of the higher-level entity in light of cheaters occurring at the lower-level [9], [ 48]. For egalitarian transitions, the second challenge poses a significant problem. This defector behavior compromises the success and survival of the group as a whole. As a result, groups that undergo egalitarian transitions must evolve mechanisms that prevent cheaters. Within this study, groups of organisms that evolved a multi-lineage solution to the problem faced the same challenge – to ensure continued group success, they needed to evolve a mechanism for preventing cheaters. Our analyses revealed the groups of organisms evolved to accomplish this objective by carefully balancing the average replication rate of genotypes to ensure rate of replication equity among the lineages within the group. These results highlight the potential for digital evolution experiments to address core evolutionary questions surrounding the major transitions in evolution.

## Materials and Methods

All experiments were conducted using the Avida digital evolution platform [27], using the deme-based grouping system. For our experiments, an Avida world consisted of 400 groups, where each group could contain up to 25 organisms. Each organism competed with others within its group for space – i.e., when an organism replicated, it replaced another organism within the group. Groups of organisms also competed for space in tournaments that occurred, in general, every 100 updates. When between-group selection occurs, tournament winners are chosen based on the range of tasks performed by organisms in the group. When between-group selection pressures are not used, tournament winners are selected at random. When we apply within-group selection pressures, the five different logic tasks are associated with varying rewards that affect the rate at which an organism reproduces within the group. Otherwise, all tasks are rewarded equally at the mean reward amount (2.8) and thus different task performance does not affect the reproductive rate of individual organisms. To study the effect of these pressures, we define four treatments: Within, which includes within-group pressures only; Between, which includes between-group pressures; Both, which includes both within- and between-group pressures; and None, a treatment that includes neither within nor between-group pressures (a control).

Relevant parameters for our experiments are summarized in Table 2. We initialized each replicate with 400 groups of digital organisms, where each group comprised 25 copies of the ancestor organism. The ancestor organism did not perform any tasks; it contains only the 12 instructions required for self-replication and 88 nop-C instructions, which perform no computation, but provide ample targets for mutation. In all cases, each treatment was replicated 30 times.

**Table 2:** Common Avida configurations used for this study.

Configuration	Value
Replicates per treatment	30
Max. population size	10,000
Number of groups	400, each a 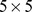 toroidal grid
Inter-tournament period length	100 updates
Tournament size	5 groups
Copy mutation rate	0.0075 (0.0003) (per instruction)
Insertion mutation rate	0.05 (0.002) (per replication)
Deletion mutation rate	0.05 (0.002) (per replication)

## Supporting Information

Figure S1 **Varying tournament size.** The mean number of different types of tasks performed by groups of organisms, where treatments had different tournament competition sizes. The maximum number of different types of tasks that can be performed by a group (indicated by a black horizontal line) is 5. Each treatment included 30 replicates.
(TIF)

Figure S2
**Varying reward displacement.** Each treatment has a different reward displacement ranging from 1/2 (all tasks are punished) to 4 (all tasks are highly rewarded). The maximum number of different types of tasks that can be performed by a group (indicated by a black horizontal line) is 5. In general, when organisms accrue an individual benefit for performing a task (i.e., the reward is 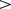
1), then the groups of organisms perform a greater diversity of types of tasks.(TIF)

Figure S3 **Varying reward distribution.** Treatments vary the distribution of the rewards among the five tasks. The maximum number of different types of tasks that can be performed by a group (indicated by a black horizontal line) is 5. In general, varying the distribution does not appreciably affect performance.
(TIF)

Figure S4 **The first twenty instructions for genotypes A and B.** Visually inspecting the differences between the genomes provides a flavor of the genetic variation present within the group.
(TIF)

Figure S5 **A visual depiction of the genotype and phenotypes of the case study.** Each square represents an organism in the group, where the shading represents the genotype and the text describes the task performed. Blank squares are organisms that did not perform a task during the analysis period.
(TIF)

Table S1 **The performance of groups of organisms when competed under inter-tournament competition periods different than the one in which they evolved.** In general, the number of unique tasks performed by group of organisms decreased.
(TEX)

Text S1 **Tournament size.** An analysis of the effect of varying tournament competition sizes.
(PDF)

Text S2 **Antagonistic Multilevel Selection Pressures.** Additional experimental results that vary the degree of antagonism between the between-group and within-group pressures.
(PDF)

Text S3 **Multi-Lineage Group Case Study.** An analysis of one highly fit group of organisms that cumulatively perform all five tasks and also exhibit genetic variation.
(PDF)

Text S4 **Single-Lineage Group Case Study.** An analysis of one highly fit group of organisms that cumulatively perform all five tasks, but all share the same genotype.
(PDF)
