# Supplementary figures and images for: The Effect of Conflicting Pressures on the Evolution of Division of Labor
Source: PLoS One. 2014 Aug 5;9(8):e102713. doi: 10.1371/journal.pone.0102713 (PMC4122366; doi:10.1371/journal.pone.0102713)

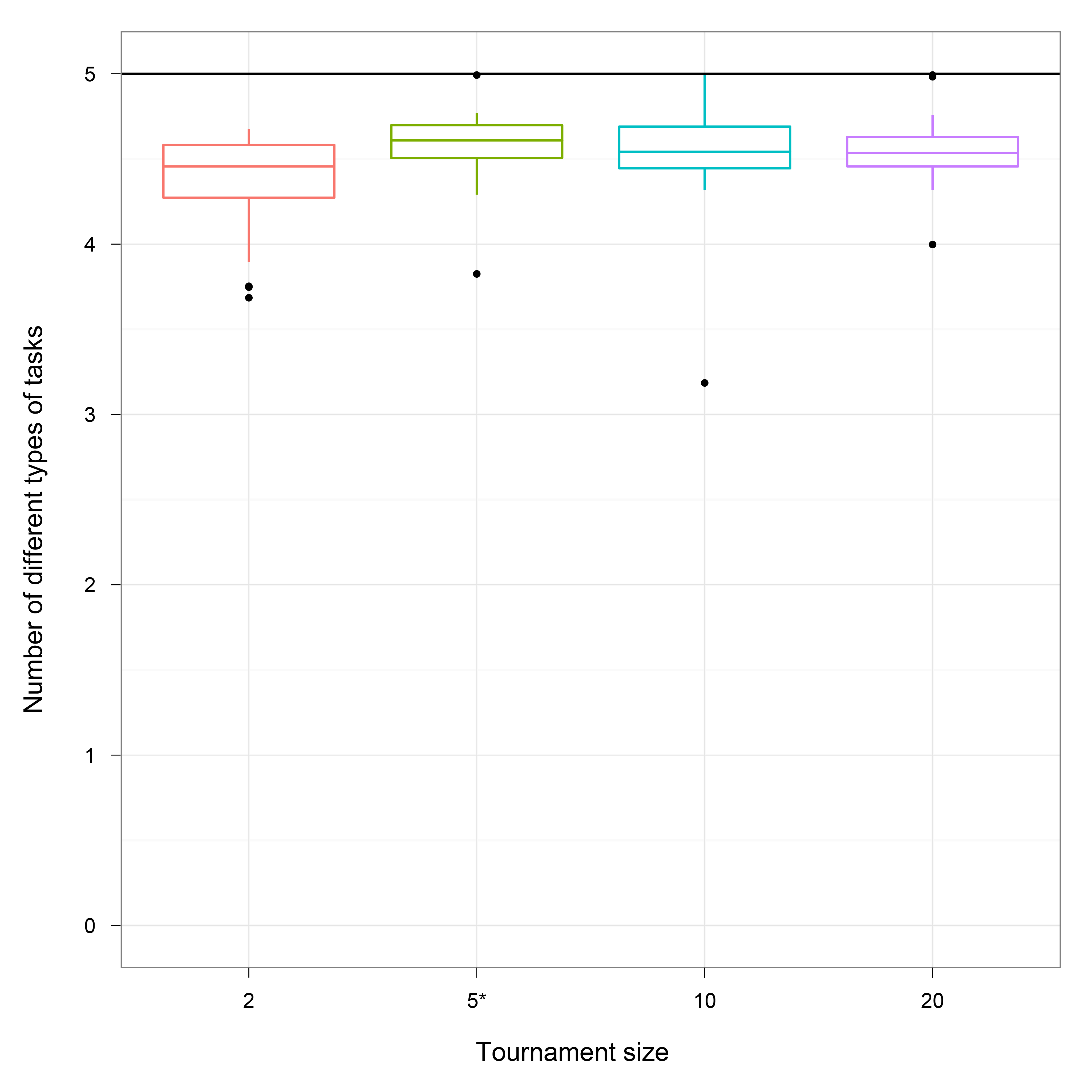

Supplement: Figure S1 — Varying tournament size. The mean number of different types of tasks performed by groups of organisms, where treatments had different tournament competition sizes. The maximum number of different types of tasks that can be performed by a group (indicated by a black horizontal line) is 5. Each treatment included 30 replicates. (TIF) [file pone.0102713.s001.tif]

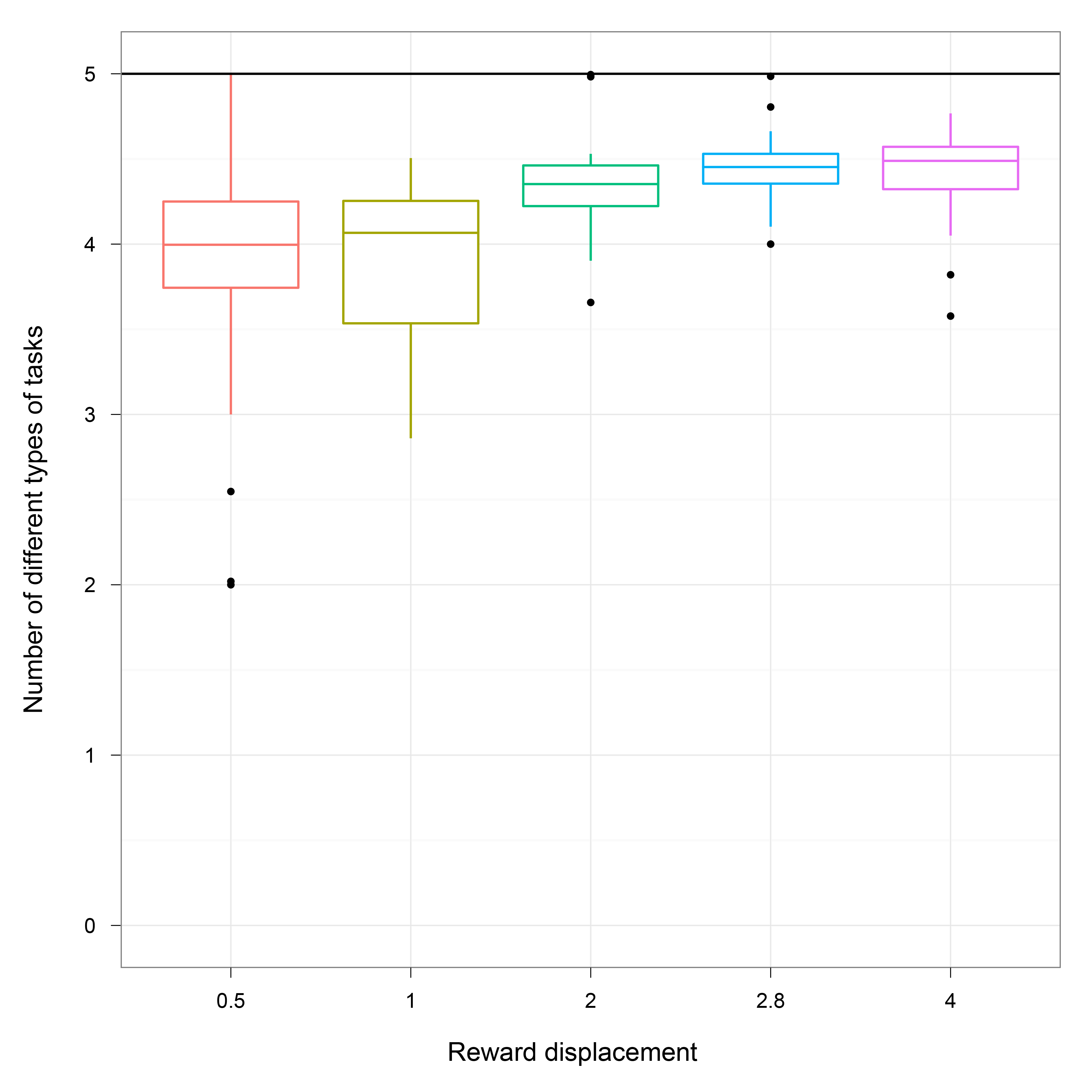

Supplement: Figure S2 — Varying reward displacement. Each treatment has a different reward displacement ranging from 1/2 (all tasks are punished) to 4 (all tasks are highly rewarded). The maximum number of different types of tasks that can be performed by a group (indicated by a black horizontal line) is 5. In general, when organisms accrue an individual benefit for performing a task (i.e., the reward is 1), then the groups of organisms perform a greater diversity of types of tasks. (TIF) [file pone.0102713.s002.tif]

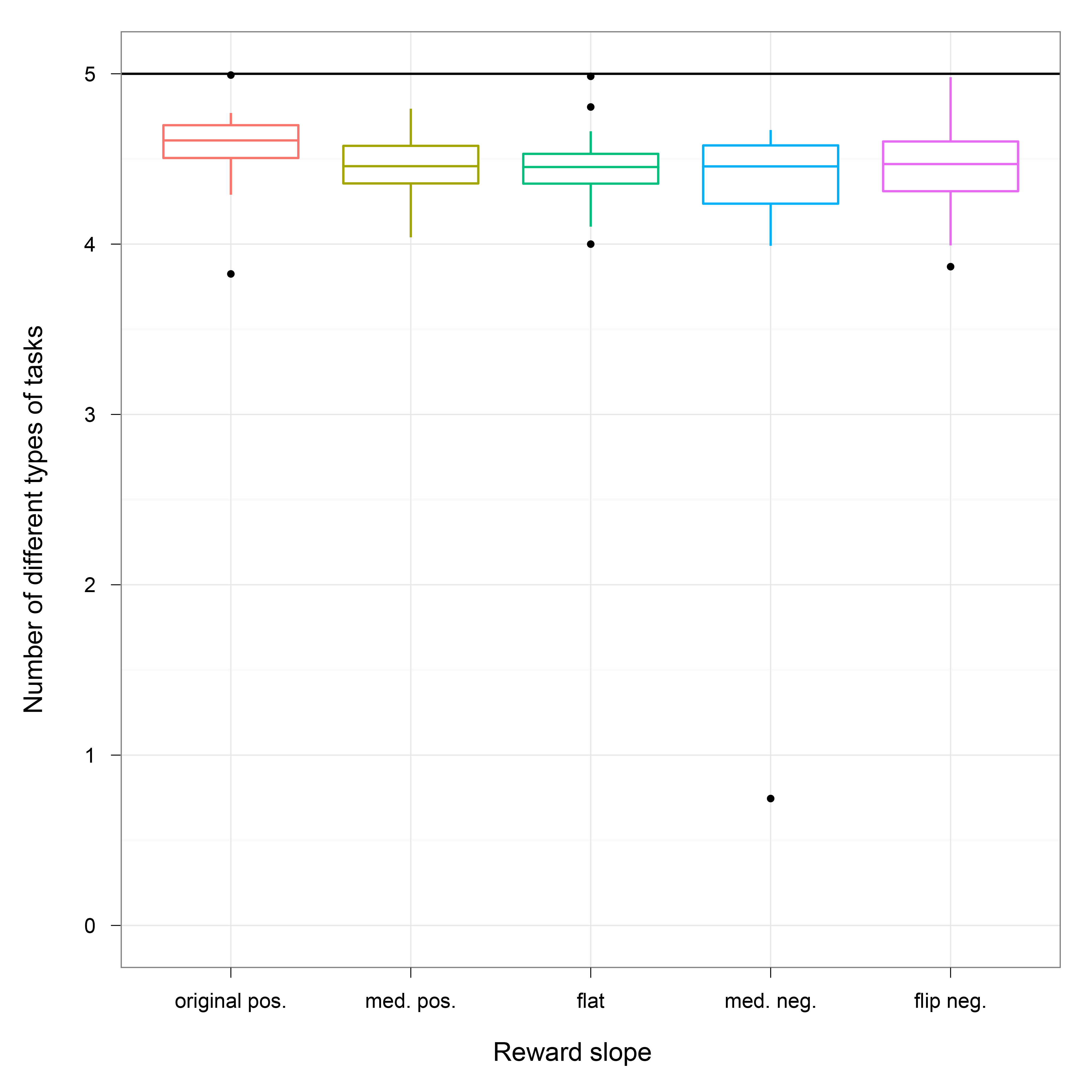

Supplement: Figure S3 — Varying reward distribution. Treatments vary the distribution of the rewards among the five tasks. The maximum number of different types of tasks that can be performed by a group (indicated by a black horizontal line) is 5. In general, varying the distribution does not appreciably affect performance. (TIF) [file pone.0102713.s003.tif]

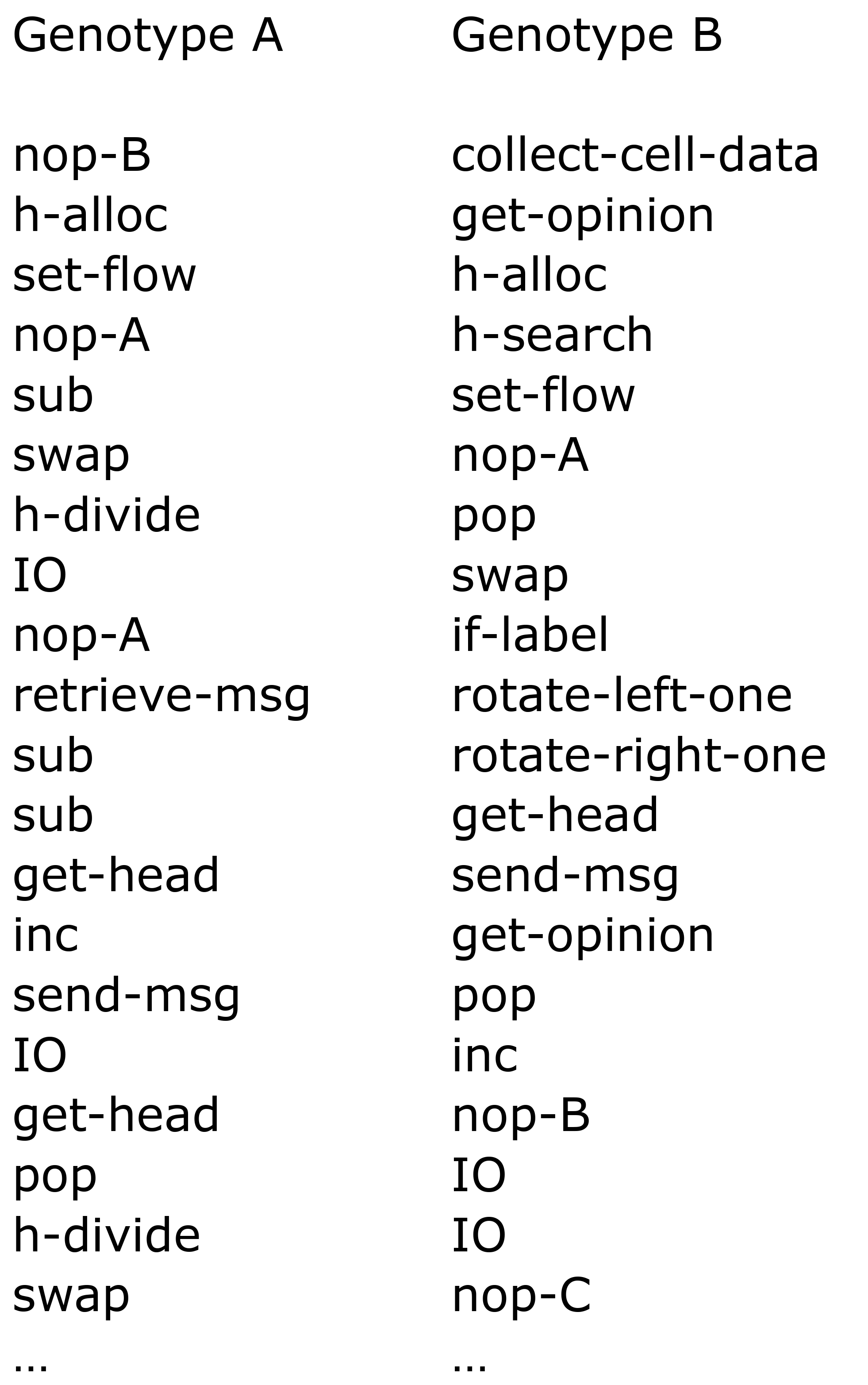

Supplement: Figure S4 — The first twenty instructions for genotypes A and B. Visually inspecting the differences between the genomes provides a flavor of the genetic variation present within the group. (TIF) [file pone.0102713.s004.tif]

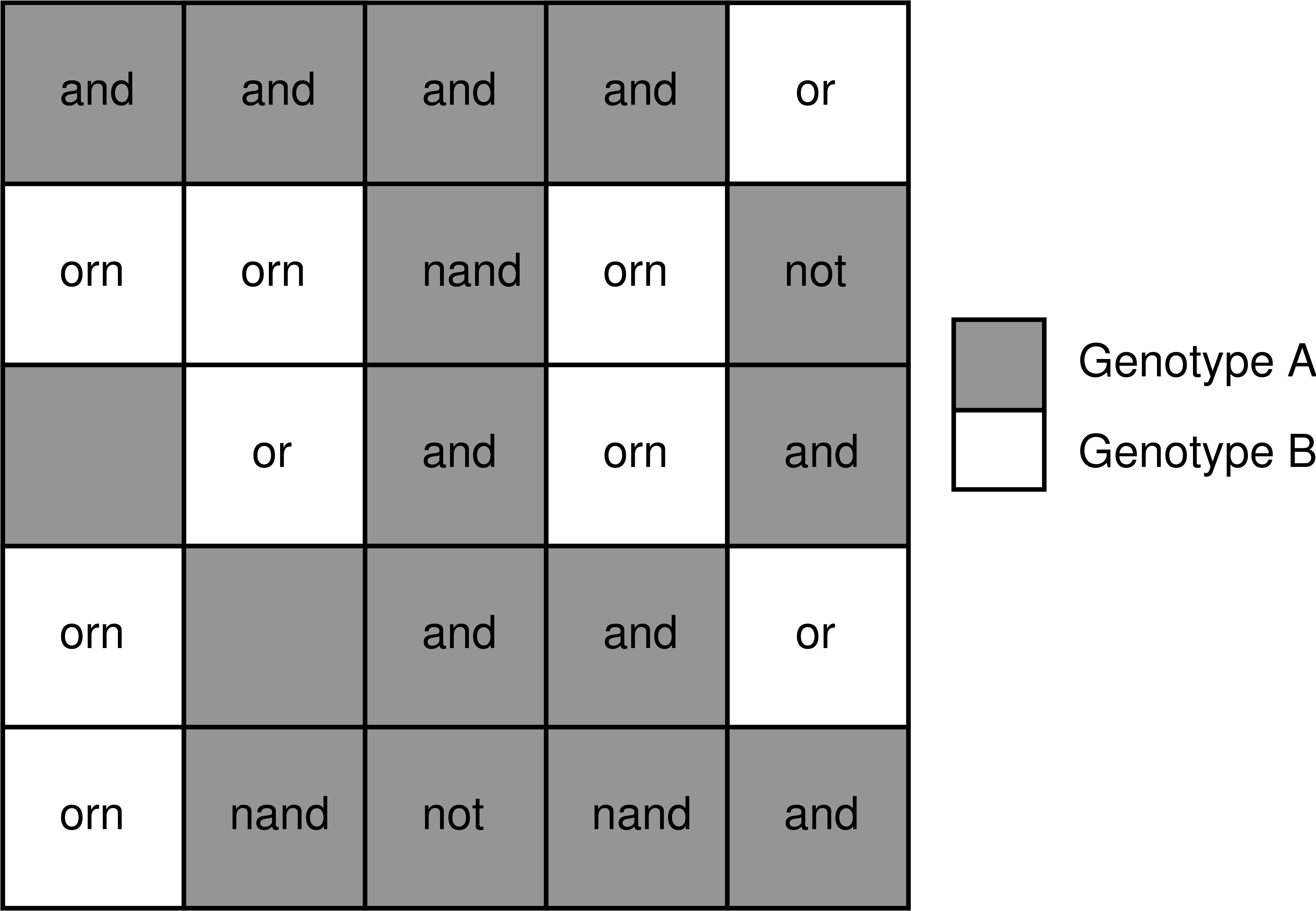

Supplement: Figure S5 — A visual depiction of the genotype and phenotypes of the case study. Each square represents an organism in the group, where the shading represents the genotype and the text describes the task performed. Blank squares are organisms that did not perform a task during the analysis period. (TIF) [file pone.0102713.s005.tif]
