# Supplementary material for: The Effect of Conflicting Pressures on the Evolution of Division of Labor
Source: PLoS One. 2014 Aug 5;9(8):e102713. doi: 10.1371/journal.pone.0102713 (PMC4122366; doi:10.1371/journal.pone.0102713)
Supplement: Text S1 — Tournament size. An analysis of the effect of varying tournament competition sizes. (PDF) [file pone.0102713.s007.pdf]

Text S1: Tournament Size Each tournament comprises 400 competitions, where each competition is used to select one group for the subsequent group-generation. A larger tournament size results in a stronger between-group selective pressure. For the experiments described in this paper, we used a tournament of size 5. To understand how tournament size affected the amount of division of labor evolved by groups of organisms that experienced both within-group and between-group selective pressures, we performed experiments with tournament sizes of 2, 10, and 20.

Figure S1 depicts the grand mean number of unique tasks of our original experiment and the three other tournament size treatments. At the final point in the experiment, the mean number of unique roles performed by the treatments were: size-2: 4.370 ± 0.052; size-5: 4.509 ± 0.062; size-10: 4.526 ± 0.055; size-20: 4.545 ± 0.034. No results are statistically significantly different from one another (Kruskal-Wallis multiple comparison, $p > 0.005$). These results indicate that, for these experiments, tournament size is not an important factor.
