# Supplementary material for: The Effect of Conflicting Pressures on the Evolution of Division of Labor
Source: PLoS One. 2014 Aug 5;9(8):e102713. doi: 10.1371/journal.pone.0102713 (PMC4122366; doi:10.1371/journal.pone.0102713)
Supplement: Text S2 — Antagonistic Multilevel Selection Pressures. Additional experimental results that vary the degree of antagonism between the between-group and within-group pressures. (PDF) [file pone.0102713.s008.pdf]

Text S2: Antagonistic Multilevel Selection Pressures

To explore how the degree of antagonism between the between-group and within-group pressures affects the amount of division of labor exhibited by groups, we performed several additional treatments that vary two key factors.

First, we studied the effect of *reward displacement*, which is how much an organism that performs a task is rewarded or punished compared to an organism that does not perform a task. We performed five additional treatments that rewarded all tasks equally and swept the reward multiplier. The treatments are:

1/2 - performing a task is detrimental to the fitness of the organism. The within-group component of this structure is in the opposite direction of the between-group pressure.

1 - performing a task has no effect on the fitness of the organism. One side-effect of this structure is that organisms that do not perform a task may replicate over organisms that do perform a task.

2 - performing any task has a positive effect on the fitness of the organism.

2.8 - performing any task has a positive effect on the fitness of the organism. This value is the mean reward of the original treatment.

4 - performing any task has a positive effect on the fitness of the organism. We selected this value to see if increasing the reward would change the speed at which organisms accrued tasks.

Results are presented in the Figure S2. In general, treatments with higher rewards evolved to perform the tasks earlier in evolutionary time. The overall end performance of the treatment depends on whether the reward displacement was negative, neutral, or positive. Specifically, the positive treatments (2, 2.8, and 4) perform more tasks than the negative and neutral treatments (Kruskal-Wallis multiple comparison, $p = 0.05$; $\frac{1}{2}$ is significantly different than 2, 2.8, and 4; 1 is significantly different than 2.8 and 4).

Second, we examined whether the *distribution* of the rewards across tasks affected the number of different types of tasks group members evolved to perform. For these treatments, the mean reward is held constant at 2.8, however, we vary how the rewards are allocated across the five tasks. Specifically, we modify the slope of the line created by connecting the values of the rewards. There are 5 different treatments:

original pos. - `NOT` (2), `NAND` (2), `AND` (3), `ORN` (3), and `OR` (4).

med. pos. - `NOT` (2.27), `NAND` (2.27), `AND` (2.93), `ORN` (2.93), and `OR` (3.6). This reward structure has a positive slope (i.e., more complex tasks are rewarded' more than less complex tasks), but the disparity among task rewards is reduced (i.e., the difference between the reward of the most and least rewarded tasks is 1.33 as opposed to 2 in the original treatment).

flat - `NOT` (2.8), `NAND` (2.8), `AND` (2.8), `ORN` (2.8), and `OR` (2.8). This reward structure provides the same reward for all tasks. The results from this treatment are also depicted in the displacement experiment as the "2.8" treatment.

med. neg. - `NOT` (3.6), `NAND` (2.93), `AND` (2.93), `ORN` (2.27), and `OR` (2.27). This reward structure has a negative slope (i.e., more complex tasks are rewarded less than simpler tasks), but the disparity among task rewards is reduced (i.e., the difference between the reward of the most and least rewarded tasks is 1.33 as opposed to 2 in the original treatment). This treatment is the flip of the med. pos treatment.

flip neg. - `NOT` (4), `NAND` (3), `AND` (3), `ORN` (2), and `OR` (2). This reward structure simply flips the original structure, i.e., the less complex tasks are rewarded more than the more complex tasks.

Results are depicted in Figure S3. In general, the distribution of the rewards among the tasks does not substantially affect the results of the treatments (Kruskal-Wallis multiple comparison, $p > 0.05$, indicating there were no significant differences).
