# Supplementary material for: The Effect of Conflicting Pressures on the Evolution of Division of Labor
Source: PLoS One. 2014 Aug 5;9(8):e102713. doi: 10.1371/journal.pone.0102713 (PMC4122366; doi:10.1371/journal.pone.0102713)
Supplement: Text S3 — Multi-Lineage Group Case Study. An analysis of one highly fit group of organisms that cumulatively perform all five tasks and also exhibit genetic variation. (PDF) [file pone.0102713.s009.pdf]

Text S3: Multi-Lineage Group Case Study

We perform a detailed analysis of one highly fit group of organisms that cumulatively performs all five tasks and also exhibits genetic variation. After one ecological period, the members of this group performed all 5 tasks and had two distinct genotypes. These genotypes had a mean Levenshtein distance of 76.5 instructions. The first 20 instructions of each genotype are depicted in Figure S4. These genotypes differ at every locus for this portion of the genotype.

To understand the specific role of the two genotypes, we analyzed the contributions of each in isolation by filling several groups with clones of a single genotype and subjecting them to an ecological period of 100 updates. This process revealed that in isolation organisms with genotype A performed tasks `NOT`, `NAND`, and `AND` and organisms with genotype B performed task `ORN` and `OR`. Because no organism gets rewarded for more than a single task, both genotypes exhibit phenotypic plasticity such that organisms of the same genotype perform different tasks. Additionally, although the genotypes are not closely related, they have evolved to fill distinct ecological niches that enable them to exhibit division of labor when placed in a group.

Because both genotypes exhibit phenotypic plasticity, we next explored how the organisms select a task to perform. We performed knockout experiments, which demonstrated that the organisms were not using location information or communication to coordinate roles. These knockouts did reveal that the organisms had evolved to use epigenetic information passed from parent to offspring to coordinate task allocation. Specifically, an organism with genotype A performed task `AND` and then stored the solution to task `NAND` in the special purpose register. After replicating, its offspring used the contents of the register to perform `NAND` and then stored the results for task `NOT` in the register. When this organism replicated, its offspring (the grand-offspring of the original organism) retrieved the contents of the register to perform `NOT`. Genotype B used the special purpose register in a similar fashion so that the parent performed `ORN` and the offspring performed task `OR`. Intriguingly, this behavior is very similar to cellular differentiation in which parent cells pass state information to offspring cells, which they use to become increasingly specialized over time.

To understand the behavior of the group as a whole, we further analyzed its behavior during one ecological period. Figure S5 depicts the genotypes and the task the organisms performed during the ecological period. During this period, all 5 tasks were performed. Overall, the two genotypes present in the group fill mutualistic ecological niches. Each genotype performed more than one type of task that

the other did not perform. When organisms with different genotypes were placed in a group together, all five types of tasks were performed.
