# Supplementary material for: The Effect of Conflicting Pressures on the Evolution of Division of Labor
Source: PLoS One. 2014 Aug 5;9(8):e102713. doi: 10.1371/journal.pone.0102713 (PMC4122366; doi:10.1371/journal.pone.0102713)
Supplement: Text S4 — Single-Lineage Group Case Study. An analysis of one highly fit group of organisms that cumulatively perform all five tasks, but all share the same genotype. (PDF) [file pone.0102713.s010.pdf]

Text S4: Single-Lineage Group Case Study

Many of the groups of organisms evolved within this study exhibited a single-lineage strategy for performing division of labor. Specifically, the genetic diversity within these groups was extremely low and the organisms used either stochasticity or phenotypic plasticity to differentiate roles. Within this section, we examine how organisms within one such single-lineage group (evolved under the `Both` treatment) managed to perform all five different tasks.

After an ecological period, the group contained only one genotype. Preliminary knockout analyses indicated that organisms of this genotype did not make use of messaging to differentiate roles. In studying the behavior of the organisms present within this group, we discovered that the genotype contained a complicated algorithm that made use of both location information and epigenetic information. Specifically, the genome of an Avida organism can contain both replication structures and task-performing structures. In this case, the two types of structures interacted in a regulatory fashion. The original organisms placed in the group used their location (i.e., whether their x coordinate was less than their y coordinate) to determine whether they should copy one instruction of their genome to an offspring earlier within their life cycle. This timing change within their replicative structure modified which task they performed – some performed `ORNOT` and some performed `NAND`.

Subsequent organisms made use of epigenetic information from their parent. Specifically, an organism whose parent performed `ORNOT` used this information to perform `AND`. The organism's offspring then used epigenetic information to perform `OR`. Similarly, an organism whose parent performed `NAND` used task information stored by the parent to perform `NOT`. Thus the same algorithm was used to perform all five of the tasks.
